# Supplementary material for: A primate nigrostriatal atlas of neuronal vulnerability and resilience in a model of Parkinson’s disease
Source: Nat Commun. 2023 Nov 18;14:7497. doi: 10.1038/s41467-023-43213-2 (PMC10657376; doi:10.1038/s41467-023-43213-2)
Supplement: Supplementary file 1 — Supplementary Information [file 41467_2023_43213_MOESM1_ESM.pdf]

A primate nigrostriatal atlas of neuronal vulnerability and resilience in a model of Parkinson's disease

Lei Tang[1,6], Nana Xu[1,6], Mengyao Huang[1,6], Wei Yi[1,6], Xuan Sang[1,6], Mingting Shao[1], Ye Li[1], Zhao-zhe Hao[1], Ruifeng Liu[1], Yuhui Shen[1], Feng Yue[2,3], Xialin Liu[1*], Chuan Xu[4*], Sheng Liu[1,5,*]

Affiliations

[1] State Key Laboratory of Ophthalmology, Zhongshan Ophthalmic Center, Sun Yat-sen University, Guangdong Provincial Key Laboratory of Ophthalmology and Visual Science, Guangzhou , China

[2] State key laboratory of digital medical engineering, School of Biomedical Engineering, Hainan University, Haikou, 570228, China

[3] Key Laboratory of Biomedical Engineering of Hainan Province, One Health Institute, Hainan University. Haikou 570228, China.

[4] Wellcome Sanger Institute, Wellcome Genome Campus, Hinxton, Cambridge, UK

[5] Guangdong Province Key Laboratory of Brain Function and Disease, Guangzhou, China

[6] These authors contributed equally

*Correspondence:

liuxl28@mail.sysu.edu.cn

cx1@sanger.ac.uk (C.X.)

liush87@mail.sysu.edu.cn (S.L.)

Supplementary figures 1-12

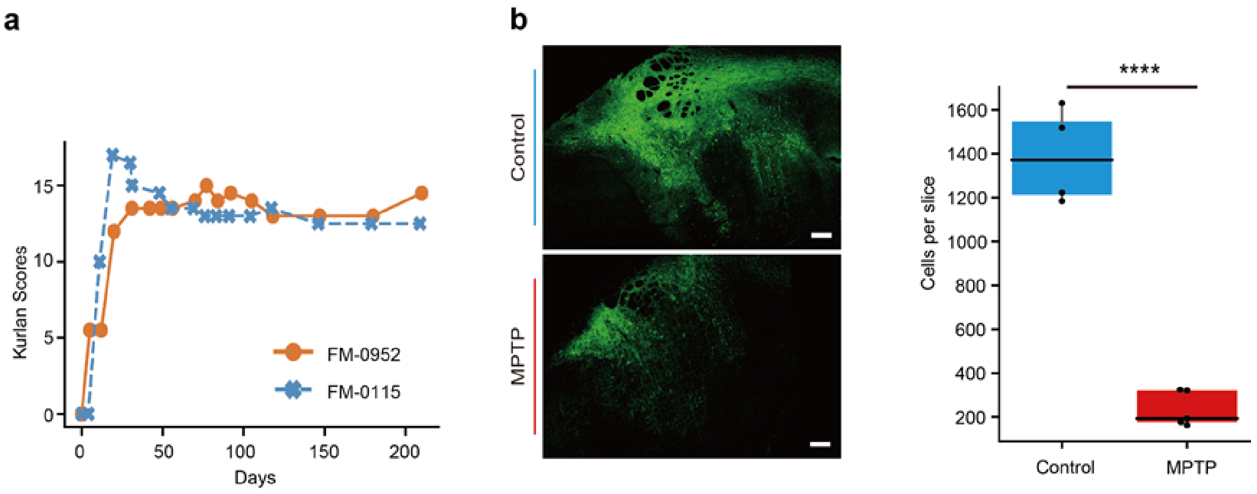

Supplementary Figure 1: Loss of dopaminergic neurons in the macaque model of parkinsonism. a Temporal pattern of the Kurlan scores after MPTP injection. Shown are patterns for two MPTP-treated macaques sampled across 17 time points. Kurlan scores after day 210 became stable and were not recorded. **b** IHC images showing TH stainings in the substantia nigra of control and MPTP-treated macaques. Scale bar, 500 μm. Box plot to the right displays the numbers of TH-positive cells per slice between control and parkinsonian macaques, with the box frames extended from the lower quartile, median, to upper quartile. ****, $P < 0.0001$, as determined by two-sided t-test. $n = 4$ samples. Source data are provided as a Source Data file.

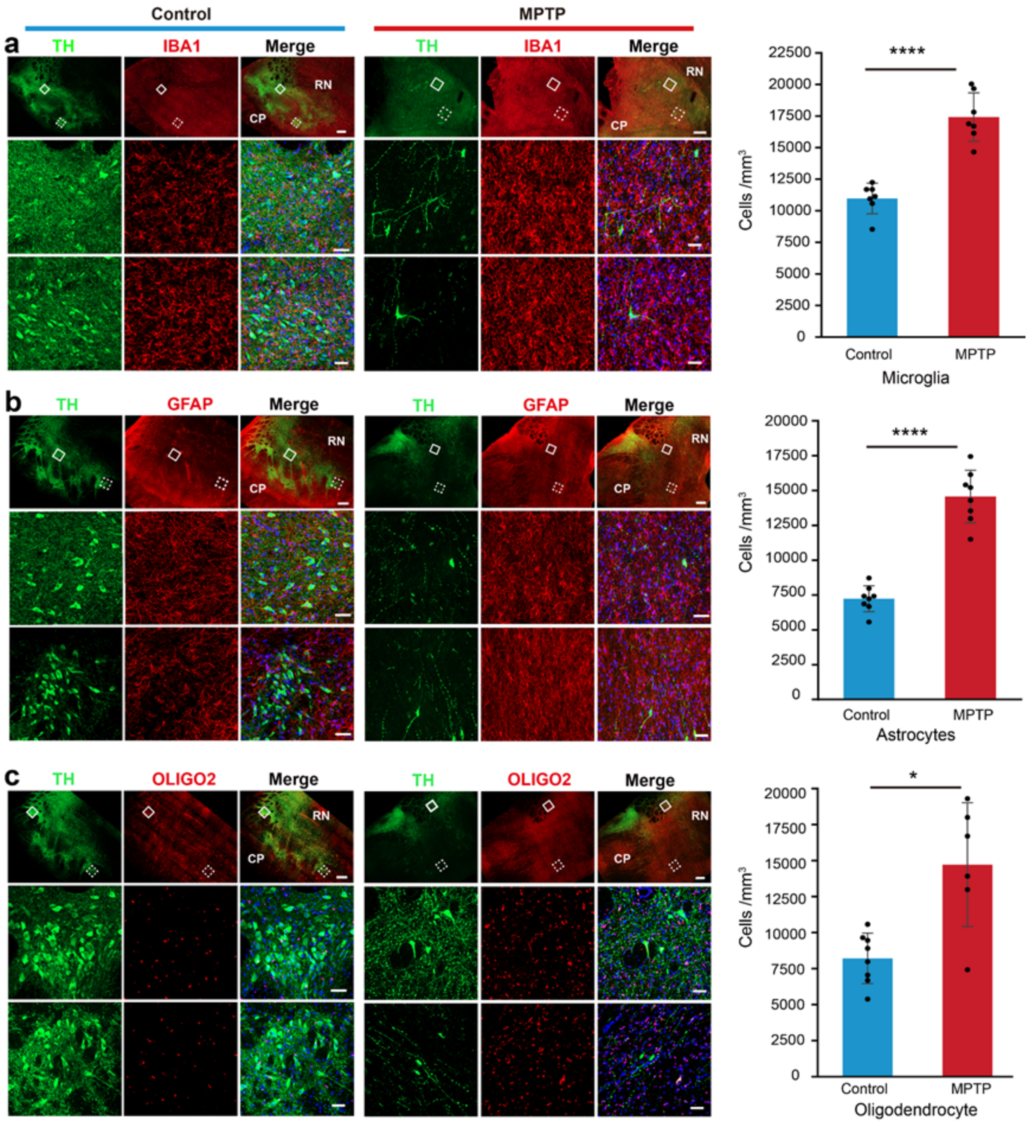

Supplementary Figure 2: Increased gliosis in the macaque model of parkinsonism. IHC images showing stainings of microglia marker IBA1 (**a**), astrocyte marker GFAP (**b**), and oligodendrocyte marker OLIGO2 (**c**) in control (left) and MPTP-treated (right) macaques in substantia nigra. Solid and dotted rectangles mark the areas corresponding to the second and third rows of the images, respectively. Bar plots to the right displays the numbers of IBA1-, GFAP-, OLIGO2-positive cells per mm^3 between control and parkinsonian macaques. Data are presented as mean ± SD. n = 7 (**a**), 8 (**b**), 8 (**c**) control samples. n = 7 (**a**), 8 (**b**), 6 (**c**) MPTP-treated samples. Scale bars: 500 µm (low magnification), 50 µm (high magnification). ****, $P < 0.0001$; *, $P < 0.05$, as determined by two-sided t-test. Source data are provided as a Source Data file.

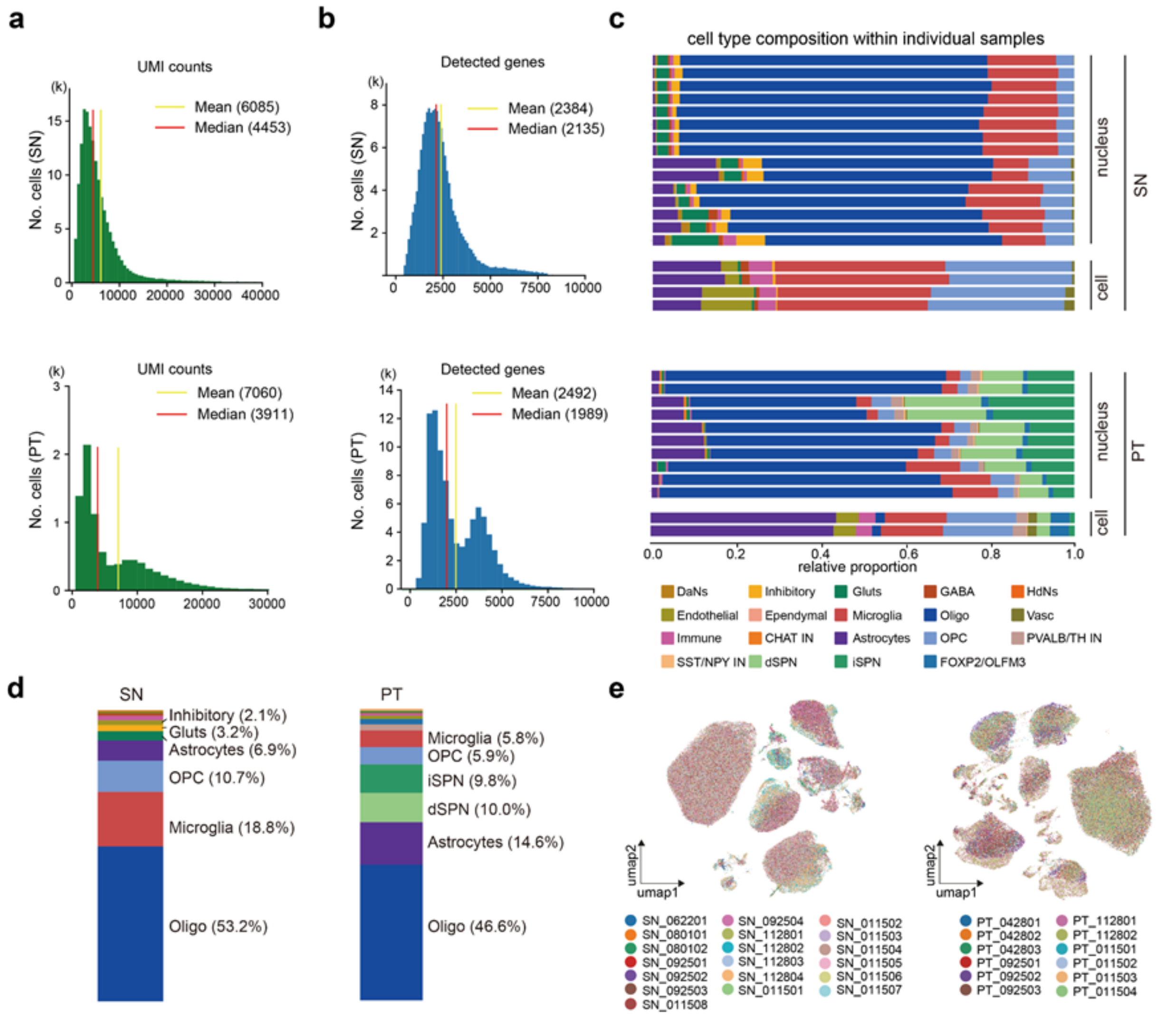

Supplementary Figure 3: Data quality control. a Distributions of UMI counts in cells of SN (top) and PT (bottom). **b** Distribution of detected genes in cells of SN (top) and PT (bottom). **c** Stacked bar plots showing the relative compositions of cell types present across brain samples. Shown are SN (top two panels) and PT (bottom two panels) samples profiled using single-nucleus (upper) and single-cell (lower) RNA-sequencing. **d** Stacked bar plots summarizing cell type compositions in the SN (left) and PT (right). **e** UMAP visualizations of SN (left) and PT (right) cells colored by sample information. SN, substantia nigra; PT, putamen. Source data are provided as a Source Data file.

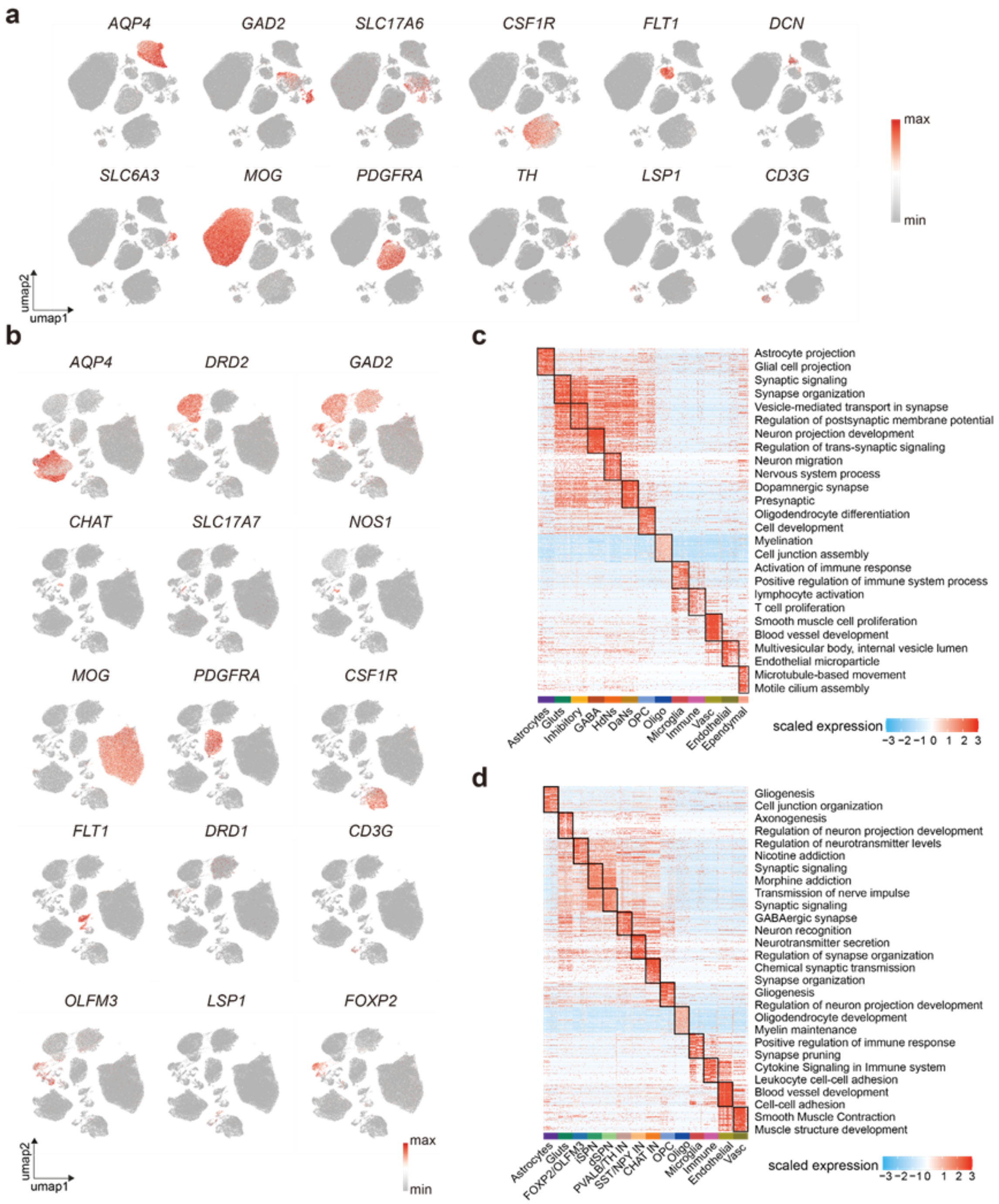

Supplementary Figure 4: Molecular signatures of SN and PT cells. a UMAP visualizations of SN cells colored by known marker genes of cell types. **b** As in **a**, but for PT cells. **c** Heatmap showing enriched functional terms across cell populations in the SN. **d** As in **c**, but for PT. SN, substantia nigra; PT, putamen.

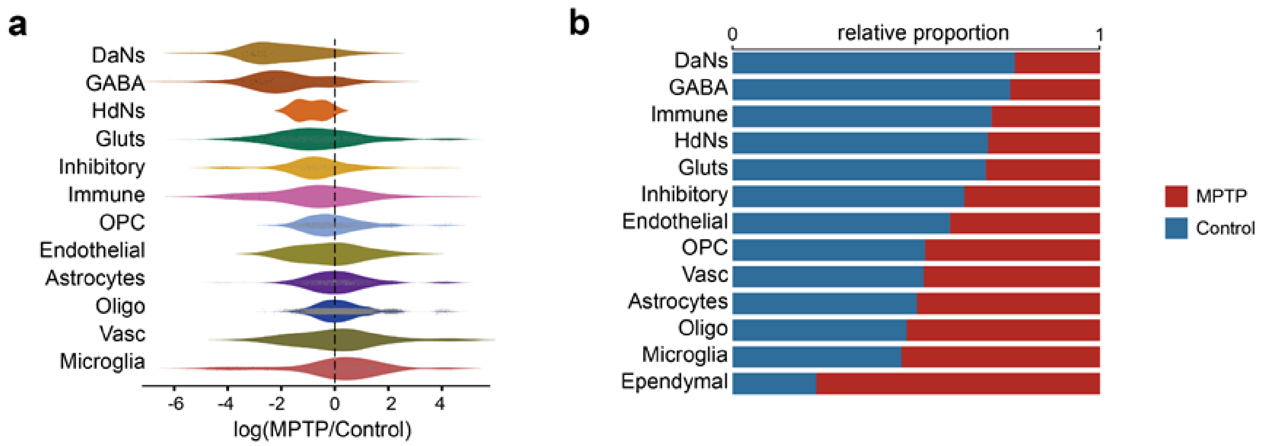

Supplementary Figure 5: Differential abundance of cell types between health and PD. a Violin plot showing cell abundance changes between parkinsonian and control subjects, with the dots representing neighborhoods grouped by cell types. **b** Bar plot showing the relative contributions of healthy (blue) and parkinsonian (red) cells to each major cell type identified. Source data are provided as a Source Data file.

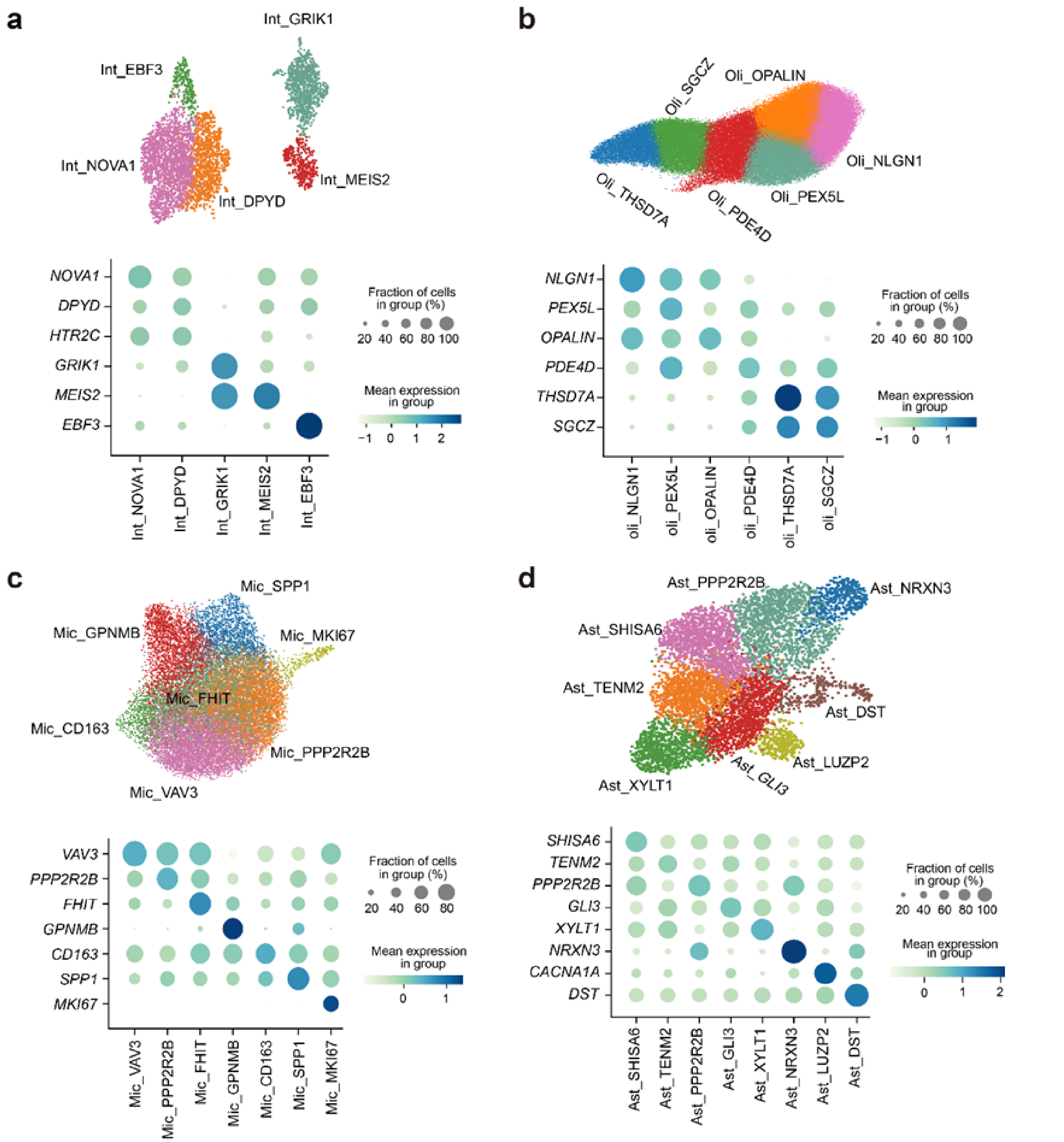

Supplementary Figure 6: Cell subtypes of inhibitory neurons and glia. a Top: UMAP visualization of inhibitory neuronal subtypes. Bottom: dot plot showing expression of selected marker genes across these subtypes. Color of the dot represents normalized gene expression, and size represents the percentage of cells expressing a given gene. **b-d** As in **a**, but for oligodendrocytes (**b**), microglia (**c**), and astrocytes (**d**).

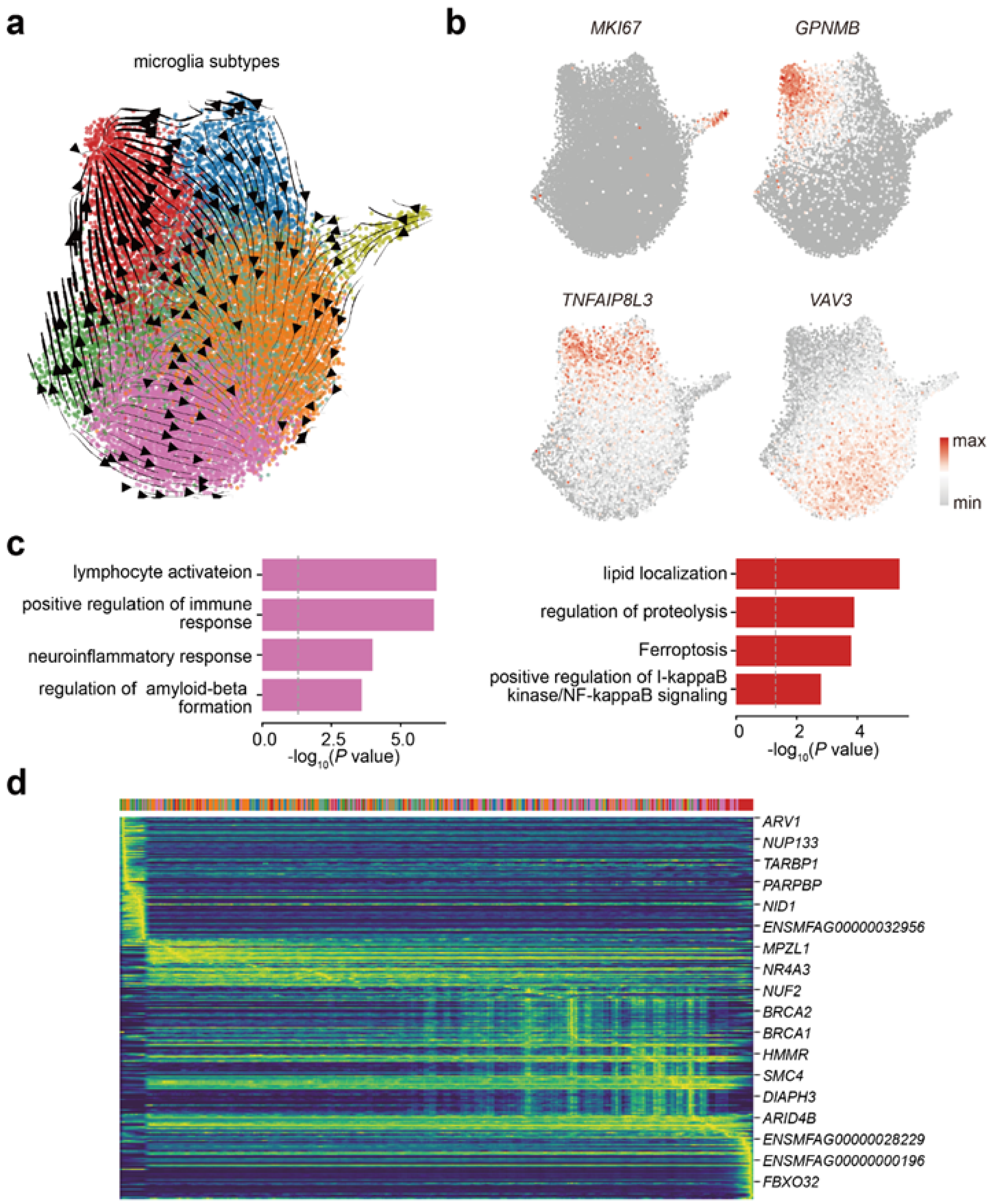

Supplementary Figure 7: RNA velocity analysis of microglial subtypes. a Dynamical model-based RNA velocities of microglia visualized as streamlines based on the UMAP embedding. **b** UMAP visualizations of microglial subtypes colored by selected marker genes. **c** Enriched gene ontology terms in activated microglial subclusters of Mic_VAV3 (left) and Mic_GPNMB (right). **d** Heatmap showing the gene expression dynamics along the latent time. Top 300 likelihood-ranked genes are selected. Source data are provided as a Source Data file

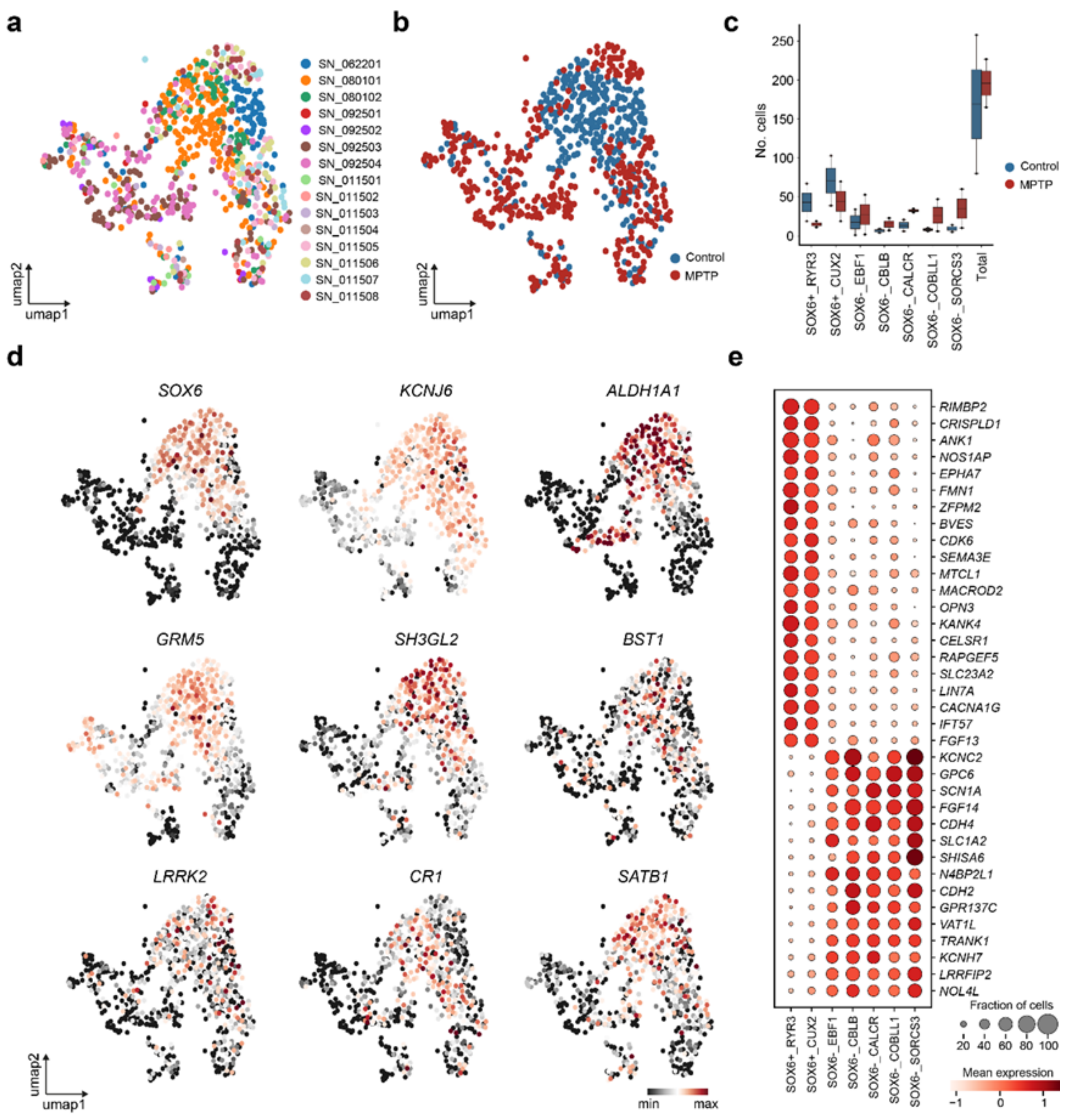

Supplementary Figure 8: Expression features of DaNs subclusters. a UMAP visualization of DaNs subclusters colored by samples. **b** UMAP visualization of DaNs subclusters colored by disease conditions. **c** Box plot displaying the numbers of healthy and parkinsonian cells across DaNs subclusters. The rightmost box plot denotes the distribution for all DaNs cells. **d** UMAP visualizations of DaNs colored by selected susceptibility genes. **e** Dot plot showing expression of vulnerability and resilience marker genes across DaNs subclusters, with color and size of the dot representing normalized gene expression and percentage of cells expressing a given gene, respectively. Source data are provided as a Source Data file.

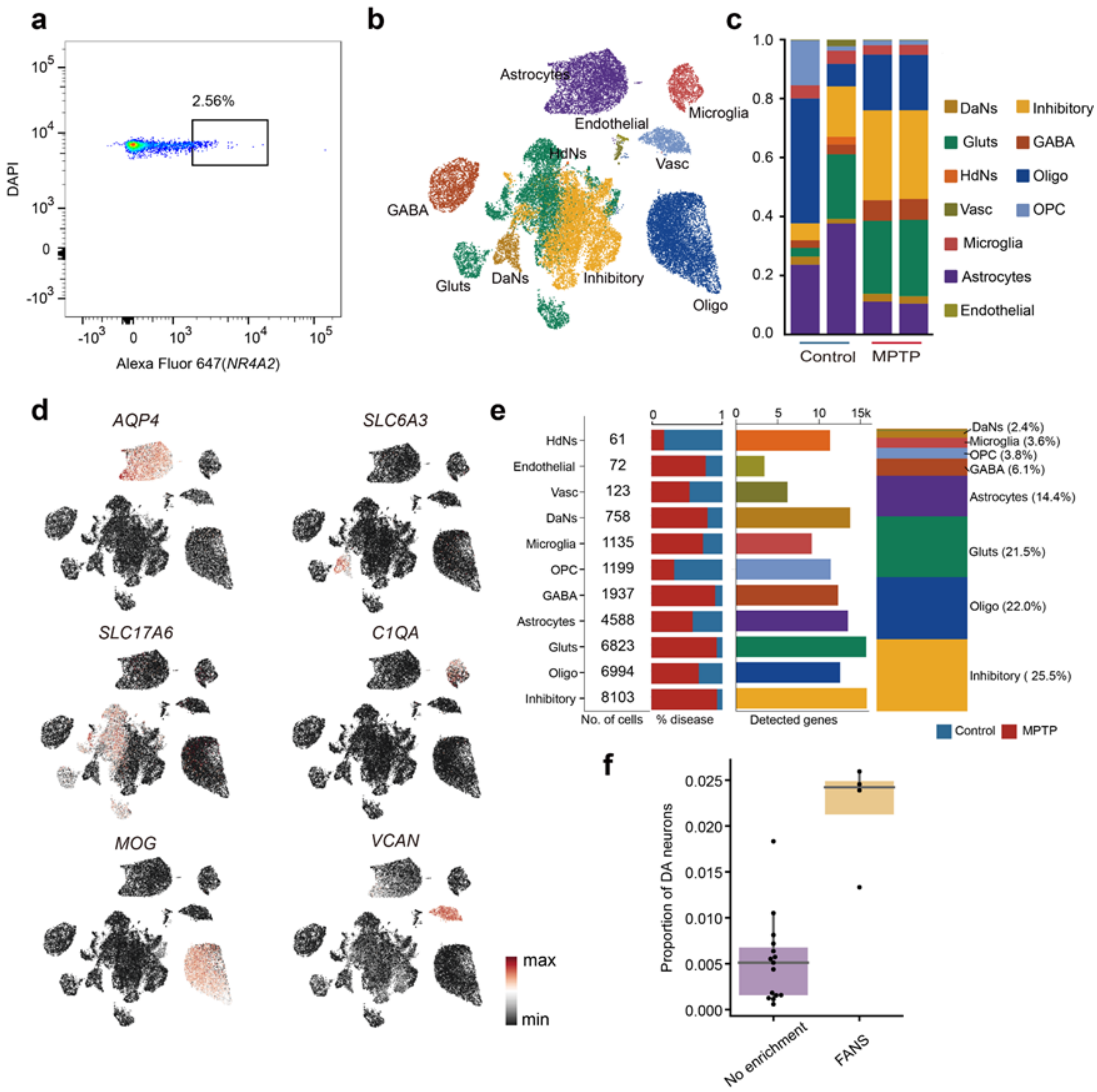

Supplementary Figure 9: Nuclei sorted from the substantia nigra of parkinsonian and healthy macaques. **a** Representative FANS plot for enriching midbrain DaNs. The NR4A2 gate was thresholded to select the top 2.3–4.0% of all nuclei. **b** UMAP visualization of all sorted nuclei colored by cell types identified. **c** Stacked bar plots showing the compositions of cell types present across macaque samples. **d** As in **b**, but colored by expression of selected marker genes. **e** Left: stacked bar plot showing the relative contributions of disease conditions to each cell type. Middle: bar plot exhibiting the number of detected genes in each cell type. Right: stacked bar plot summarizing cell type compositions of the sorted nuclei. **f** Box plot showing proportions of DaNs per replicate for FANS enriched and unbiased samples. Source data are provided as a Source Data file.

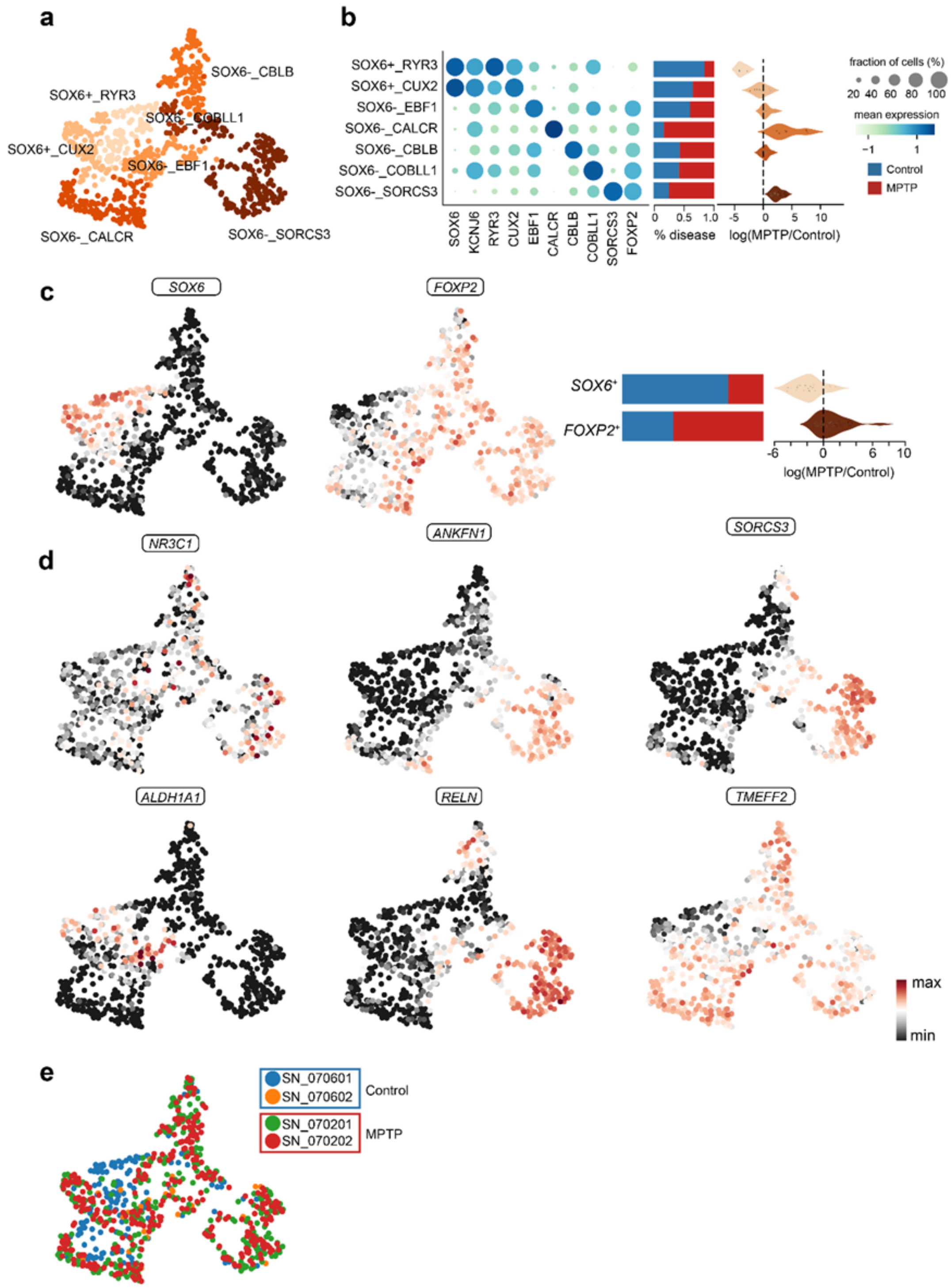

Supplementary Figure 10: Cellular and molecular diversity of sorted dopaminergic neurons in macaque SN. a UMAP visualization of DaNs subclusters colored by cell subtype labels transferred from the snRNA-seq dataset. **b** Left: dot plot showing expression of representative marker genes across DaNs subtypes. Color of the dot represents normalized gene

expression, and size represents the percentage of cells expressing a given gene. Middle: stacked bar plot exhibiting the proportions of cells in normal (blue) and parkinsonian (red) conditions. Right: violin plot showing cell abundance changes between parkinsonian and control subjects, with the dots representing neighborhoods grouped by cell subtypes. **c** UMAP visualization of DaNs colored by expression of *SOX6* and *FOXP2*. Violin to the right shows cell abundance changes between two neuronal groups (*SOX6*$^{+}$ versus *FOXP2*$^{+}$). **d** UMAP visualization of DaNs colored by expression of selected genes within the FOXP2 and NR3C1 regulons. **e** UMAP visualization of DaNs subclusters colored by sample information. Source data are provided as a Source Data file.

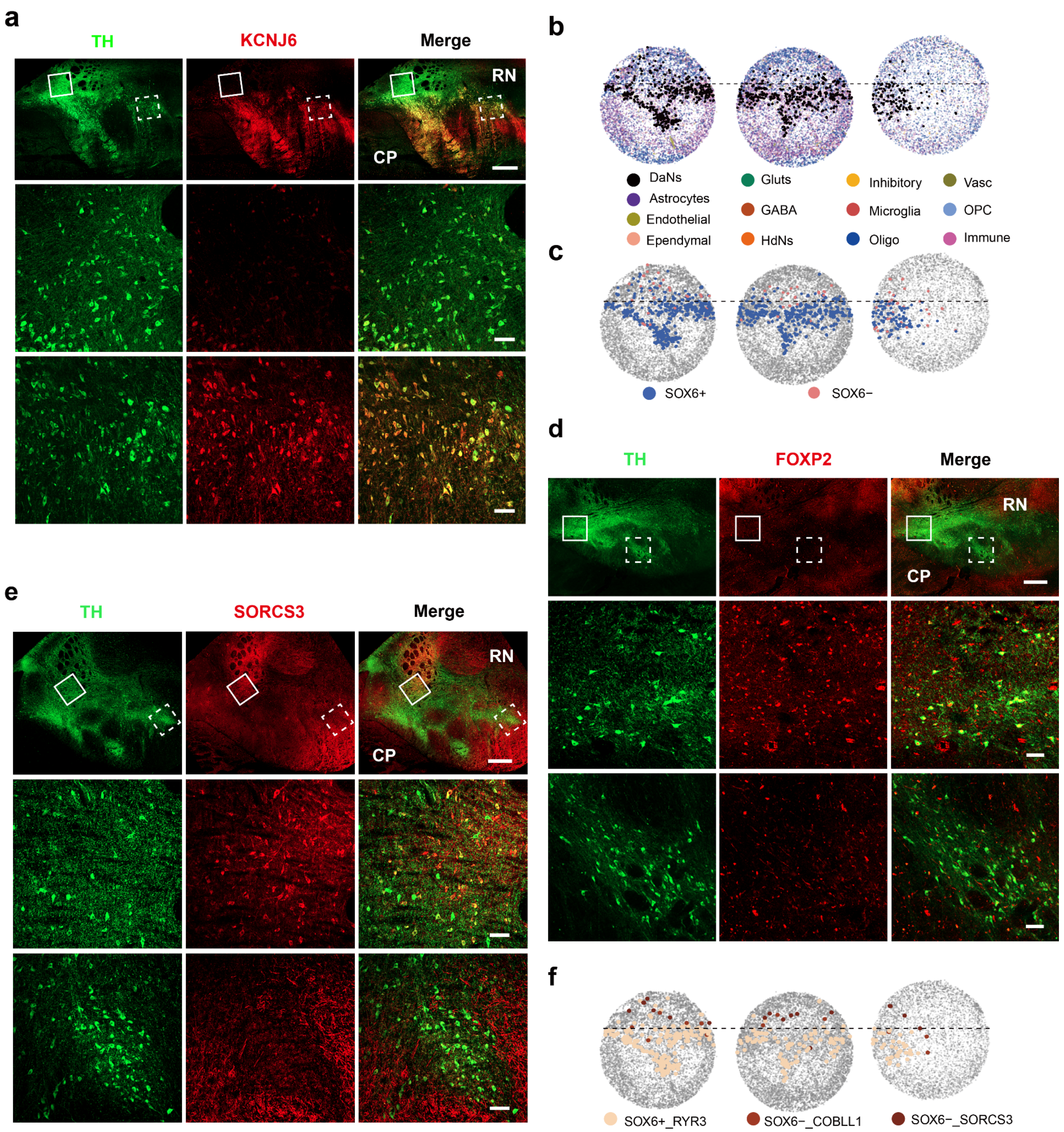

Supplementary Figure 11: Spatial distributions of dopaminergic neurons. a IHC images showing co-stainings of the PD-vulnerable protein KCNJ6 (red) and the general dopaminergic neuronal marker TH (green), as well as merged images. Solid and dotted rectangles mark the areas corresponding to the second and third rows of the images, respectively. Scale bar represents 1000 um in the whole SN and 100 um for zoomed in areas. **b,c** The distribution of cell types deconvoluted from spatial transcriptomic beads corresponding to major cell types (b), *SOX6*- and

SOX6^{+} subtypes (c). **d,e** As in **a**, but for the resilience markers FOXP2 (d) and SORCS3 (e). **f** As in **c**, but for the most vulnerable (SOX6+_RYR3) and resilient (SOX6-_COBLL1 and SOX6-_SPRCS3) DaNs subtypes.

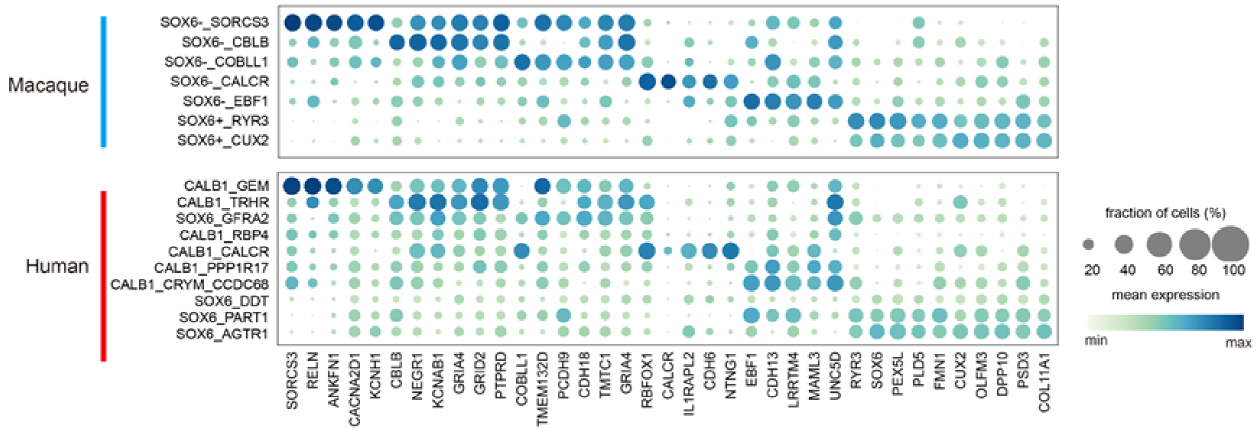

Supplementary Figure 12: Expression of marker genes in human and macaque DaNs. Dot plots showing expression of selected marker genes in macaque (top) and human (bottom) DaNs. Color of the dot represents normalized gene expression, and size represents the percentage of cells expressing a given gene.
