# Supplementary material for: A primate nigrostriatal atlas of neuronal vulnerability and resilience in a model of Parkinson’s disease
Source: Nat Commun. 2023 Nov 18;14:7497. doi: 10.1038/s41467-023-43213-2 (PMC10657376; doi:10.1038/s41467-023-43213-2)
Supplement: Supplementary file 3 — Description of Additional Supplementary Files [file 41467_2023_43213_MOESM3_ESM.docx]

**Description of Additional Supplementary Files**

**Supplementary Data 1. Basic information of the macaques.**

Age, sex, experimental group, and cell extraction method for macaques used in this study.

**Supplementary Data 2. PD and AD risk genes reported in previous publications.**

PD and AD risk genes identified from previous genome-wide association studies.

**Supplementary Data 3. Activated gene regulatory networks in dopaminergic neurons.**

Regulon scores of dopaminergic neuronal clusters calculated by pySENIC.

**Supplementary Data 4. Differentially expressed genes between the MPTP-treated and control macaques for each DaNs subcluster.**

Differentially expressed genes were calculated by a Wilcoxon Rank Sum test, with the resulting p-values adjusted by Bonferroni correction.

**Supplementary Data 5. Marker genes of resistant DaNs and Gluts.**

Marker genes of the resistant DaNs and Gluts subtypes were calculated by a Wilcoxon Rank Sum test, with the resulting p-values adjusted by Bonferroni correction.

**Supplementary Data 6. Shared upregulated genes in MPTP-treated macaques for glial cells between SN and PT.**

Differentially expressed genes were calculated by a Wilcoxon Rank Sum test, with the resulting p-values adjusted by Bonferroni correction.

**Supplementary Data 7. Region-specific upregulated genes in MPTP-treated macaques for non-neuronal clusters between SN and PT.**

Differentially expressed genes were calculated by a Wilcoxon Rank Sum test, with the resulting p-values adjusted by Bonferroni correction.

**Supplementary Data 8. Kurlan scores of the MPTP-treated macaques in this study.**

Kurlan scores of the two MPTP-treated macaques from MPTP injection to 210 days after injection.
