# Supplementary material for: A primate nigrostriatal atlas of neuronal vulnerability and resilience in a model of Parkinson’s disease
Source: Nat Commun. 2023 Nov 18;14:7497. doi: 10.1038/s41467-023-43213-2 (PMC10657376; doi:10.1038/s41467-023-43213-2)
Supplement: Supplementary file 12 — Reporting Summary [file 41467_2023_43213_MOESM12_ESM.pdf]

nature portfolio

Corresponding author(s): Xialin Liu, Chuan Xu, Sheng Liu

Last updated by author(s): 2023.10.11

Reporting Summary

Nature Portfolio wishes to improve the reproducibility of the work that we publish. This form provides structure for consistency and transparency in reporting. For further information on Nature Portfolio policies, see our Editorial Policies and the Editorial Policy Checklist.

Statistics

For all statistical analyses, confirm that the following items are present in the figure legend, table legend, main text, or Methods section.

n/a	Confirmed	
☐	☒	The exact sample size (*n*) for each experimental group/condition, given as a discrete number and unit of measurement
☐	☒	A statement on whether measurements were taken from distinct samples or whether the same sample was measured repeatedly
☐	☒	The statistical test(s) used AND whether they are one- or two-sided<br>*Only common tests should be described solely by name; describe more complex techniques in the Methods section.*
☒	☐	A description of all covariates tested
☐	☒	A description of any assumptions or corrections, such as tests of normality and adjustment for multiple comparisons
☐	☒	A full description of the statistical parameters including central tendency (e.g. means) or other basic estimates (e.g. regression coefficient) AND variation (e.g. standard deviation) or associated estimates of uncertainty (e.g. confidence intervals)
☐	☒	For null hypothesis testing, the test statistic (e.g. *F*, *t*, *r*) with confidence intervals, effect sizes, degrees of freedom and *P* value noted<br>*Give P values as exact values whenever suitable.*
☒	☐	For Bayesian analysis, information on the choice of priors and Markov chain Monte Carlo settings
☒	☐	For hierarchical and complex designs, identification of the appropriate level for tests and full reporting of outcomes
☐	☒	Estimates of effect sizes (e.g. Cohen's *d*, Pearson's *r*), indicating how they were calculated

Our web collection on statistics for biologists contains articles on many of the points above.

Software and code

Policy information about availability of computer code

Data collection	Illumina Novaseq 6000 and 10x Genomics with Chromium Controller Readliness Test were used for single cell sequencing.
Data analysis	Single cell RNA-sequencing data analysis using Cell ranger v.6.0 (10x Genomics), Scrublet 0.2.1, Scanpy 1.8.2, pySCENIC 0.11.2, GRNBoost2, Harmony 1.0, RNA velocyto 0.17.15, and scVelo 0.2.2, SCCAF 0.0.10. Gene network visualized by Cytoscape 3.9.1. Images were processed and misanalysed using Zeiss ZEN software suites(v23) and Adobe Photoshop software. Customized source code is available at https://github.com/ leitang607 /Parkinson_disease

For manuscripts utilizing custom algorithms or software that are central to the research but not yet described in published literature, software must be made available to editors and reviewers. We strongly encourage code deposition in a community repository (e.g. GitHub). See the Nature Portfolio guidelines for submitting code & software for further information.

Data

Policy information about availability of data

All manuscripts must include a data availability statement. This statement should provide the following information, where applicable:

- Accession codes, unique identifiers, or web links for publicly available datasets
- A description of any restrictions on data availability
- For clinical datasets or third party data, please ensure that the statement adheres to our policy

The raw single cell RNA sequencing data in this paper are publicly available at the EMBL-EBI with the accessible links https://www.ebi.ac.uk/biostudies/

arrayexpress/studies/ through the accession code E-MTAB-13437. Public datasets utilized in this study are available in the Gene Expression Omnibus database under accession codes GSE178265, GSE152058, and GSE116470. All data supporting the findings of this study are provided within the paper and its Supplementary information. Source data are provided with this paper.

Research involving human participants, their data, or biological material

Policy information about studies with human participants or human data. See also policy information about sex, gender (identity/presentation), and sexual orientation and race, ethnicity and racism.

Reporting on sex and gender	n/a
Reporting on race, ethnicity, or other socially relevant groupings	n/a
Population characteristics	n/a
Recruitment	n/a
Ethics oversight	n/a

Note that full information on the approval of the study protocol must also be provided in the manuscript.

Field-specific reporting

Please select the one below that is the best fit for your research. If you are not sure, read the appropriate sections before making your selection.

☒ Life sciences ☐ Behavioural & social sciences ☐ Ecological, evolutionary & environmental sciences

For a reference copy of the document with all sections, see nature.com/documents/nr-reporting-summary-flat.pdf

Life sciences study design

All studies must disclose on these points even when the disclosure is negative.

Sample size	No statistical method was used to predetermine sample sizes. The number of animals and cells were determined to ensure the biological replicates for each cell population and the number of cells in each population met or exceed the comparable published single-cell datasets (e.g. Kamath, T. et al. 2022, Nat. Neurosci. 2022)
Data exclusions	During data analysis, some cells within each sample were excluded for the following reasons: 1) high mitogene percentage (>30%) and 2) low UMI count(< 200), and 3) clusters were identified as doublets and excluded if they had elevated doublet score and the combined marker gene expression profiles of more than one cell type. The criteria was determined based on the distribution of the datasets and applied equally to all samples
Replication	Full replication of the observations was not feasible due to the size and cost of the experiment. The observations were validated by previous work or other methodology including immunofluoscent staining. The immunostaining results were replicated with at least three animals and indicated in the figure legend. The experiments were performed based on the availability of the animals without a set frequency
Randomization	Randomization was not applicable for this study. There is no treatment or intervention to the samples. Therefore there is no need for randomization.
Blinding	Blinding was not applicable for this study. To avoid bias, all samples were treated equally with the same rigorous criteria

Reporting for specific materials, systems and methods

We require information from authors about some types of materials, experimental systems and methods used in many studies. Here, indicate whether each material, system or method listed is relevant to your study. If you are not sure if a list item applies to your research, read the appropriate section before selecting a response.

Materials & experimental systems

n/a	Involved in the study
☐	☒ Antibodies
☒	☐ Eukaryotic cell lines
☒	☐ Palaeontology and archaeology
☐	☒ Animals and other organisms
☒	☐ Clinical data
☒	☐ Dual use research of concern
☒	☐ Plants

Methods

n/a	Involved in the study
☒	☐ ChIP-seq
☐	☒ Flow cytometry
☒	☐ MRI-based neuroimaging

Antibodies

Antibodies used

Mouse anti-Tyrosine Hydroxylas, clone LNC1, Millipore, Cat# MAB318-AF488, 1:500 dilution
Rabbit polyclonal anti-FOXP2, Abcam, Cat# ab16046, 1: 2000 dilution
Rabbit polyclonal anti-GIRK2 (Kir3.2) , Alomone Labs, Cat#APC-006, 1:1000 dilution
Rat monoclonal anti-SorCS3, R&D, clone 339624, Cat# MAB3067, 1:200 dilution
Rabbit anti-IBA1, Fujifilm Wako, Cat# 019-19741, 1:500 dilution
Rabbit polyclonal anti OLIGO2, Merck millipore, Cat# AB9610, 1:500 dilution
Chicken polyclonal anti-Glial Fibrillary Acidic Protein, Millipore, Cat# AB5541, 1:2000 dilution

Validation

Well characterized commercial antibodies were used.

Tyrosine Hydroxylas, Millipore: Validated by the vendor with western blotting in mouse brain lysates.
https://www.merckmillipore.com/CN/zh/product/Anti-Tyrosine-Hydroxylase-Antibody-clone-LNC1,MM_NF-MAB318

FOXP2, Abcam: Validated by the vendor. Positive IHC detected in mouse brain tissue and human testis. Positive WB detected in human 293T cell lysate. Positive ICC/IF detected in HepG2 cells.
https://www.abcam.cn/products/primary-antibodies/foxp2-antibody-ab16046.html

GIRK2 (KCNJ6), Alomone Labs: Validated by the vendor. Positive IF detected in mouse and rat brain tissues. Validated in the references provided by the vendor in human ventral mesencephalic neural stem cell line 1.
https://www.alomone.com/p/anti-kir3-2-girk2/APC-006

SorCS3, R&D: Validated in the references provided by the vendor.
https://www.rndsystems.com/cn/products/human-mouse-sorcs3-antibody-339624_mab3067#product-citations

IBA1, Fujifilm Wako: Validated by the vendor with IHC in mouse Retinal, spinal and cerebellum.
https://labchem-wako.fujifilm.com/jp/product/detail/W01W0101-1974.html

OLIGP2: Millipore: Validated by the vendor with immunofluorescence staining in human, rat and mouse cell lines and tissues, western Blot in human, rat and mouse brain.

Glial Fibrillary Acidic Protein Millipore: This Anti-Glial Fibrillary Acidic Protein Antibody is validated by the vendor for use in IC, IH, IH(P), WB for the detection of GFAP. https://www.merckmillipore.com/HK/en/product/Anti-Glial-Fibrillary-Acidic-Protein-Antibody,MM_NF-AB5541

Animals and other research organisms

Policy information about studies involving animals; ARRIVE guidelines recommended for reporting animal research, and Sex and Gender in Research

Laboratory animals

We used adult macaque monkeys (Macaca fascicularis) of both sex (5 males and 3 females) at the age of 6-18 YO.

Wild animals

The study did not involve wild animals.

Reporting on sex

There are 5 male and 3 female macaques In 10x Genomics dataset. But there were no obvious difference between each sample, therefore, we did not do further research.

Field-collected samples

The study did not involve samples collected from the field.

Ethics oversight

All experimental procedures were approved by and in accordance with the Animal Care and Use Committee of Zhongshan Ophthalmic Center, Sun Yat-sen University. The study was performed in accordance with the Public Health Service Guide to the Care and Use of Laboratory Animals.

Note that full information on the approval of the study protocol must also be provided in the manuscript.

Flow Cytometry

Plots

Confirm that:

☒ The axis labels state the marker and fluorochrome used (e.g. CD4-FITC).

☒ The axis scales are clearly visible. Include numbers along axes only for bottom left plot of group (a 'group' is an analysis of identical markers).

☒ All plots are contour plots with outliers or pseudocolor plots.

☒ A numerical value for number of cells or percentage (with statistics) is provided.

Methodology

Sample preparation	All samples were prepared according to the Methods section of this paper. Nuclei isolates were obtained from Snap-frozen macaque tissue.
Instrument	Samples were flow-sorted using a BD FACSAriaFusion
Software	FlowJo™ v10 were used for flow cytometry analysis
Cell population abundance	Abundances of the NR4A2+ neurons were determined post-sorting using single-nucleus RNA-sequencing
Gating strategy	No preliminary FSC-A/SSC-A gating was performed. Single nuclei gating was FSC-H/FSC-A, DAPI+ and NR4A2+ nuclei were selected.

☒ Tick this box to confirm that a figure exemplifying the gating strategy is provided in the Supplementary Information.
